# Elevated β-Carotene Production Using Codon-Adapted CarRA&B and Metabolic Balance in Engineered *Yarrowia lipolytica*

**DOI:** 10.3389/fmicb.2021.627150

**Published:** 2021-03-04

**Authors:** Liang Liu, Yu Ling Qu, Gui Ru Dong, Jing Wang, Ching Yuan Hu, Yong Hong Meng

**Affiliations:** ^1^Engineering Research Center of High Value Utilization of Western China Fruit Resources, Ministry of Education, National Research and Development Center of Apple Processing Technology, Shaanxi Engineering Laboratory for Food Green Processing and Safety Control, College of Food Engineering and Nutritional Science, Shaanxi Normal University, Xi’an, China; ^2^Department of Human Nutrition, Food and Animal Sciences, College of Tropical Agriculture and Human Resources, University of Hawai’i at Mānoa, Honolulu, HI, United States

**Keywords:** β-carotene, metabolic balance, *CarRA*, *Yarrowia lipolytica*, *CarB*

## Abstract

β-carotene is a precursor of vitamin A and has multiple physiological functions. Producing β-carotene by microbial fermentation has attracted much attention to consumers’ preference for natural products. This study focused on improving β-carotene production by constructing codon-adapted genes and minimizing intermediate accumulation. The codon-adapted *CarRA* and *CarB* genes from the industrial strain of *Blakeslea trispora* were integrated into the genome of the *Yarrowia lipolytica* to construct YL-C0, the baseline strain for producing β-carotene. Thereafter, the β-carotene biosynthetic pathway’s metabolic balance was accurately regulated to reduce the intermediates’ accumulation. Notably, the β-carotene content increased by 21 times to reach 12.5 dry cell weight (DCW) mg/g when minimizing HMG-CoA and FPP accumulation. Further, we improved the expression levels of the *CarRA* and *CarB* genes to minimize the accumulation of phytoene and lycopene. Total production of β-carotene of 1.7 g/L and 21.6 mg/g DCW was achieved. These results reveal that the rate-limiting enzymes CarRA and CarB of *B. trispora* exhibited higher catalytic activity than the same enzymes from other microorganisms. Promoting metabolic balance by minimizing the accumulation of intermediates is a very effective strategy for increasing β-carotene. The β-carotene-producing strain constructed in this study has established the foundation for its potential use in industrial production. These successful engineering strategies also provide a foundation for large-scale production of other terpenoids.

## Introduction

β-carotene has multiple physiological functions, including antioxidant, anti-cancer, prevention of senile dementia, and vitamin A’s precursor (Yen et al., 2015; Wu et al., 2017). In recent years, the market demand for β-carotene was increasing dramatically due to its broad applications in pharmaceuticals, nutraceuticals, cosmetics, and foods (Lee and Schmidt-Dannert, 2002; Das et al., 2007; Ajikumar et al., 2008). The Global Market Insights reports that the global β-carotene market is estimated to exceed USD 500 million in 2023. With the consumers’ preference for products from natural sources, microbial fermentation has proven to be an economical, environmentally friendly, and sustainable technique for β-carotene production.

*Blakeslea trispora* is a natural producer in industrial β-carotene production (Nanou and Roukas, 2016). However, mixed strain fermentation’s complex process limits the yield of β-carotene (Mehta et al., 2003; Zhu et al., 2015). With the rapid development of metabolic engineering, heterologous genes’ expression has become a prospective strategy in model organisms, such as *Saccharomyces cerevisiae*, *Escherichia coli*, and *Yarrowia lipolytica* (Yoon et al., 2007; Olson et al., 2016). As a generally recognized-as-safe (GRAS) organism, the oleaginous yeast *Y. lipolytica* can provide enough lipid bodies for liposoluble pigment storage. More importantly, *Y. lipolytica* has abundant acetyl-CoA, which can be used as a precursor for β-carotene synthesis. Thus, *Y. lipolytica* has been considered a promising biotechnological chassis for β-carotene synthesis in metabolic engineering manipulation.

Generally, for the β-carotene biosynthesis in *Y. lipolytica*, it is required to introduce heterologous genes encoding the carotene synthesis pathway, e.g., from *Pantoea ananatis*, *Schizochytrium sp.*, *Xanthophyllomyces dendrorhous*, and *Mucor circinelloides*. However, *Y. lipolytica*’s ability to produce β-carotene differs when heterologous β-carotene synthesis genes from different microorganisms are incorporated. For example, the content of β-carotene reached 16 mg/g DCW when the genes *CrtB* and *CrtI* of *P*. *ananatis* were integrated into *Y. lipolytica*, and 0.41 mg/g DCW was obtained when *CarS* of *Schizochytrium sp.* was integrated into *Y. lipolytica* (Matthäus et al., 2014; Gao et al., 2017a). Less than 1 mg/g DCW of β-carotene was produced when the genes *CrtE*, *CrtI*, and *CrtYB* from *X. dendrorhous* were integrated into *Y. lipolytica* (Gao et al., 2014). Currently, the most prolific β-carotene-producing strain was achieved by integrating the gene *CarRP* and *CarB* from *M. circinelloides* (Larroude et al., 2017). However, when the *CarRP* and *CarB* genes from *M. circinelloides* were expressed in *Y. lipolytica*, the strain produced less β-carotene than the strain expressing the *CarRA* and *CarB* genes from *B. trispora* (Yin et al., 2017). Therefore, the catalytic activity of the *CarRA* and *CarB* of *B. trispora* should be systemically investigated to explore further the potential to improve the yield of β-carotene.

Several strategies currently used to promote the production of β-carotene include regulation of key rate-limiting enzyme expression, replenishment of energy, and optimization of culture conditions. Overexpression of the key rate-limiting enzymes is considered the most commonly used approach among these strategies because it diverts the precursor from the mevalonate pathway to β-carotene synthesis. Hydroxymethylglutaryl-CoA reductase (HMGR) is the first rate-limiting enzyme and mainly catalyzes the reaction from hydroxymethylglutaryl-CoA (HMG-CoA) to mevalonate. Overexpression of the truncated HMGR (tHMGR) produced more β-carotene than the untruncated HMGR because tHMGR is more stable than HMGR (Donald et al., 1997; Jon and Victor, 2004). The β-carotene production increased by 95–134% when one copy of tHMGR was overexpressed (Gao et al., 2017b; Larroude et al., 2017). Besides tHMGR, geranylgeranyl diphosphate synthase (GGS1) is another rate-limiting enzyme involves in the condensation reaction from farnesyl pyrophosphate (FPP) to geranylgeranyl pyrophosphate (GGPP). If the GGS1 activity is inefficient, the condensation of two FPP molecules forming GGPP is limited, which results in the shortage of precursor GGPP and prevents the flow of metabolic flux toward β-carotene production. Overexpressing GGS1 also has been used to improve carotenoid production (Verwaal et al., 2007; Gao et al., 2017b).

Nevertheless, the above studies only focused on increasing β-carotene production but did not pay attention to the metabolic balance. The metabolic balance means no accumulation of intermediates at the connecting node when combining separately engineered upstream and downstream pathways. Flux imbalance in synthetic pathways often leads to loss of carbon flow, accumulation of harmful intermediates, and formation of byproducts, which reduces product yield and growth ratio. For example, the integration of four copies of *tHmgR* resulted in a limited increase of β-carotene (Gao et al., 2017b). The excessive tHMGR causes the accumulation of mevalonate, destroying metabolic balance if it cannot be further transformed. Therefore, how to promote the pathway flux and keep a subtle balance becomes a common concern in metabolic engineering.

The objective of this study was to construct a productive engineered *Y. lipolytica* strain for producing β-carotene. First, we introduced the codon-adapted *CarRA* and *CarB* into *Y. lipolytica* to generate the baseline strain capable of producing β-carotene. Subsequently, from the perspective of metabolic balance, we precisely regulated the expression levels of key enzymes tHMGR, GGS1, CarRA, and CarB by minimizing intermediates’ accumulation to improve β-carotene content, and an excellent β-carotene-producing strain was obtained. Our results have established an efficient construction strategy for carotene biosynthesis and obtained a productive strain for potential industrial applications.

## Materials and Methods

### Strain, Medium, and Culture Conditions

The strains and plasmids used in this study are shown in Supplementary Table 1, and the primer’s sequences are listed in Supplementary Table 2. *E. coli* DH5α was used for plasmid propagation. *E. coli* DH5α was grown at 37°C with constant shaking in Luria–Brentani (LB) broth supplemented with 100 mg/mL ampicillin. For shake-flask cultivation, the engineered strains were cultivated in the yeast extract (Sangon Biotech, Shanghai, China) peptone dextrose (YPD) medium and incubated at 30°C, 180 rpm (ZC-250, Suzhou Peiying, China) in 250 mL Erlenmeyer flasks containing 50 mL fermentation medium. SD-ura medium contained 20 g/L glucose, 5 g/L (NH_4_)_2_SO_4_, 1.7 g/L yeast nitrogen base with ammonium sulfate and without amino acids, and 2 g/L uracil.

### Codon-Adapted Genes Construction

The amino acid sequences of CarRA and CarB were ascertained based on the preferred codons in *Y. lipolytica*^1^. The nucleotide sequence was compared to the codon usage preference in the *Y. lipolytica* genome using the Kazusa DNA Research Institute Database (see text footnote 1). For the same amino acid, the infrequently used codons in the *B. trispora* genome were replaced by the frequently used codons in the *Y. lipolytica* genome. The designed genes were synthesized by Genscript Biotech (Nanjing, China).

### Plasmids and Strains Construction

For gene deletion, the upstream and downstream of the *Ku70* coding regions were amplified with the primers *Ku70-*up-F/R and *Ku70*-down-F/R, respectively. The upstream region, linearized with *Apa*I and *Xba*I, was then inserted into pLoxp-ura3-Loxp (Fickers et al., 2003; Wang et al., 2016). The downstream region, linearized with *Spe*I and *Nde*I, was then inserted into pLoxp-ura3-Loxp to form the plasmid pLoxp-ura3-Loxp-Δ*Ku70*. pLoxp-ura3-Loxp-Δ*Ku70* digested with *Apa*I was transformed into *Y. lipolytica* po1f (ATCC # MYA-2613) to generate the high homologous recombination efficiency of *Y. lipolytica* po1f-Δ*Ku70*. The pLoxp-ura3-Loxp-Δ*Snf* plasmids were constructed as described for pLoxp-ura3-Loxp-Δ*Ku70*. For gene integration, the expression cassette P_*TEF*_-*CarRA*–xpr2t*-*P_*TEF*_*-CarB*-xpr2t was ligated via *Spe*I into the plasmid pLoxp-ura3-Loxp-Δ*Snf* to generate the plasmid pLoxp-ura3-Loxp-Δ*Snf*::optAB. The plasmid pLoxp-ura3-Loxp-Δ*Snf*::optAB was then transformed into *Y. lipolytica* po1f-Δ*Ku70* after digestion with *Spe*I to obtain the strain YL-C0. The plasmids pLoxp-ura3-Loxp-Δ*Lip1* was constructed as described for pLoxp-ura3-Loxp-Δ*Ku70.* The expression cassette P_*TEF*_-*tHmgR*-xpr2t-P_*TEF*_*-Ggs1*-xpr2t was ligated via *Spe*I into the plasmid pLoxp-ura3-Loxp-Δ*Lip1* to generate the plasmid pLoxp-ura3-Loxp-Δ*Lip1*:: *tHmgR-Ggs1*. The plasmid pLoxp-ura3-Loxp-Δ*Lip1*:: *tHmgR-Ggs1* was then transformed into YL-C0 after digestion with *Spe*I to obtain the engineered strain YL-C1. The plasmids pLoxp-ura3-Loxp-Δ*Pox3* was constructed as described for pLoxp-ura3-Loxp-Δ*Ku70.* The expression cassette P_*TEF*_-*tHmgR*-xpr2t, P_*TEF*_-2*tHmgR*-xpr2t, P_*TEF*_-*tHmgR-*2*Ggs1*-xpr2t, P_*TEF*_-*tHmgR-*2*Ggs1*-xpr2t were ligated via *Spe*I into the plasmid pLoxp-ura3-Loxp-Δ*Pox3* to generate the plasmids pLoxp-ura3-Loxp-Δ*Pox3*::*tHmgR*, pLoxp-ura3-Loxp-Δ*Pox3*::2*tHmgR*, pLoxp-ura3-Loxp-Δ*Pox3*::*tHmgR-Ggs1*, and pLoxp-ura3-Loxp-Δ*Pox3*::*tHmgR-*2*Ggs1*. The plasmids pLoxp-ura3-Loxp-Δ*Pox3*::*tHmgR*, pLoxp-ura3-Loxp-Δ*Pox3*::2*tHmgR*, pLoxp-ura3-Loxp-Δ*Pox3*::*tHmgR-Ggs1*, and pLoxp-ura3-Loxp-Δ*Pox3*::*tHmgR-*2*Ggs1* were then, respectively, transformed into YL-C1 after digestion with *Spe*I to obtain the strain YL-C2, YL-C3, YL-C4, and YL-C5.

The plasmids pLoxp-ura3-Loxp-Δ*Pox4* was constructed as described for pLoxp-ura3-Loxp-Δ*Ku70*. The expression cassette P_*TEF*_-*CarRA*-xpr2t*-*P_*TEF*_*-CarB*-xpr2t was ligated via *Spe*I into the plasmid pLoxp-ura3-Loxp-Δ*Pox4* to generate the plasmid pLoxp-ura3-Loxp-Δ*Pox4*::optAB. The plasmid pLoxp-ura3-Loxp-Δ*Pox4*::optAB was then transformed into YL-C5 after digestion with *Spe*I to obtain the engineered strain YL-C6.

### Transformation

*Yarrowia lipolytica* transformation was performed with Zymogen Frozen EZ Yeast Transformation Kit II (Zymo Research, Irvine, CA, United States). For gene deletion and integration, approximately 0.5–1 μg of linearized DNA was used for transformation, and then 150–200 μL of transformation mixture was plated on SD-ura solid media. The marker ura3 was removed as described previously (Fickers et al., 2003; Wang et al., 2016). Selection plates were incubated at 30°C for 2–4 days. Diagnostic PCR and DNA sequencing were used to confirm gene deletion and gene integration. All primers used for identification of the engineered strains are listed in Supplementary Table 2.

### Bioreactor Fermentation

Bioreactor fermentation was completed in a 5-L baffled stirred-tank bioreactor with a medium containing 10 g/L glucose, 10 g/L yeast extract powder, 10 g/L casein peptone, 3 g/L (NH_4_)_2_SO_4_, 2.5 g/L KH_2_PO_4_, and 0.5 g/L MgSO_4._ After inoculation, the glucose was maintained at around 10 g/L by continuously supplemented in fed-batch fermentation. Oxygen was supplied in filtered air at 1–5 L/min, cascading agitation between 400 and 1000 rpm to maintain pO2 levels at 15–20% until the air input flow and agitation reached the maximum value of 5 L/min and 1000 rpm, respectively. The temperature was maintained at 30°C, and the culture’s pH was continuously controlled at 5.50 using 15% ammonia.

### β-Carotene Measurement

Dry cell weight was measured with an analytical balance. In brief, the cells were harvested by centrifugation at 4000 *g*, 4°C for 5 min, and then the cell pellet was washed three times using sterile water and dried at 80°C to a constant weight (50 mL of fermentation broth). The β-carotene content was analyzed as described with slight modifications (Gao et al., 2017b). A 1 mL sample was used to extract β-carotene by centrifuging at 4000 *g* for 5 min, resuspended in 1 mL 3 M HCl with vortexing for 8 min, incubated for 5 min at 100°C, and then cooled in an ice bath for 5 min. The sample was washed once with distilled water, and the mixture was vortexed for 8 min after 1 mL acetone was added until the pellet was colorless. Subsequently, the extracts collected by centrifuging at 5000 *g* for 5 min and filtered with a 0.45 μm pore size filter were used for high-performance liquid chromatography (HPLC, U3000, Thermo Fisher Scientific, Waltham, MA, United States) analysis. The HPLC was equipped with a C_18_ column (4.6 mm × 250 mm), and UV/VIS signals were detected at 450 nm. The mobile phase consisted of methanol-acetonitrile-isopropanol (3:5:2 v/v/v) with a flow rate of 0.8 mL/min at 25°C. The β-carotene standard (Sigma-Aldrich; St. Louis, MO, United States) was solved in acetone to prepare standard curves.

### HMG-CoA Content Quantification

Hydroxymethylglutaryl-CoA was analyzed using HMG-CoA Enzyme-Linked Immunosorbent Assay (ELISA) Reagent Kit (Mbbiology Biological, Jiangsu, China), which uses a double-antibody sandwich assay to determine the level of HMG-COA in the specimen. The microplate was coated with purified HMG-CoA antibody to prepare a solid-phase antibody, and HMG-CoA was sequentially added to the microwell of the coated monoclonal antibody, followed by a combination with HRP-labeled HMG-CoA antibody to form an antibody-antigen-enzyme-labeled antibody complex, which is thoroughly washed and then plated with tetramethylbenzidine (TMB). TMB is converted to blue under the catalysis of the HRP enzyme and converted to the final yellow color by the action of acid. The color depth is positively correlated with HMG-COA in the sample. The HMG-COA was determined according to the manufacturer’s protocol with slight modifications. In brief, cells (1 mL of fermentation broth) were harvested at 96 h by centrifuging at 4000 *g* for 5 min, washed twice with PBS, broken with liquid nitrogen grounding method, resuspended in 1 mL PBS. The sample was then obtained after 5 min centrifugation at 4000 *g*. 10 μL sample, and 40 μL sample diluent were added to the ELISA plate, incubated at 37°C for 30 min, washed five times with washing solution after discarding the liquid. Then 50 μL conjugate reagent was added and followed by incubation at 37°C for 30 min, and the sample was rewashed five times with a washing solution. Subsequently, 50 μL chromogenic agent A and 50 μL chromogenic agent B was added to the sample, and the mixture was incubated in the dark at 37°C for 10 min. The reaction was stopped using 50 μL stop solution. The HMG-CoA was measured using ELISA analytical instruments (SpectraMax 190, Thermo; CA, United States) at 450 nm.

### FPP Content Quantification

The FPP can be dephosphorylated by pyrophosphatase and alkaline phosphatase to form the corresponding farnesol, which can be isolated and quantified by GC-MS. The content of FPP was determined using published method (Huang et al., 2011) with slight modifications. Cells (100 mL of fermentation broth) were harvested at 96 h by centrifuging at 4000 *g*, 4°C for 5 min, washed twice with PBS, broken with liquid nitrogen grounding method, resuspended in 3 mL buffer (1 M diethanolamine, 0.5 mM MgCl_2_, pH 9.8). The supernatant was then collected by centrifuging at 4000 *g*, 4°C for 5 min, followed by the addition of pyrophosphatase (3U) (Sigma-Aldrich; St. Louis, Mo, United States) incubated at 25°C for 1 h. Subsequently, alkaline phosphatase (3U) (Sigma-Aldrich) was added to the sample obtained above, incubated at 37°C for 1 h, and extracted using 1 ml hexane. The mixture was vortexed for 5 min, centrifuged at 12000 *g* for 20 min, and then the hexane phase was analyzed using GC-MS (QP2010UItra, Shimadzu, Kyoto, Japan) equipped with a capillary column RTX-5MS (30 m × 0.25 mm × 0.25 mm). FPP production (1 μL) was maintained at 100°C for 2 min, heated to 300°C at 10°C/min, and left to stand at 300°C for 10 min. Mass spectrometer operating conditions were: electron impact energy 70 eV; emission current 250 μA, transfer line 310°C, ion source temperature 250°C, scan rate 0.3 scans / s, and mass range 25–800 Da.

### Quantitative PCR (qPCR) Analysis

Transcriptional levels of the related genes in the β-carotene synthesis pathway were determined by qPCR. Total RNA was extracted according to the manufacturer’s protocol (Transgen; Beijing, China). The qPCR was performed using the SYBR tip green qPCR supermix kit (Transgen; Beijing, China). The *Actin* gene was used as the internal control; the relative gene expression analysis was performed using the method published previously (Su et al., 2018).

### Statistics Analysis

All experiments were repeated three times, and all values are expressed as the mean ± standard deviation. The statistical analysis of data and plots was performed using Origin software when necessary. Data in Figures 3–5 were analyzed using one-way ANOVA, and LSD was used to separate the means.

Accession numbers: opt *CarRA* (KY971027), opt *CarB* (KY971026), *GGS1* (YALI0D17050g), *HMGR* (YALI0E04807g), *Ku70* (YALI0C08701p), *Snf* (YALI0D02101g), *Lip* (YALI0E10659g), *Pox3* (YALI0D24750g), *Pox4* (YALI0E27654g).

## Results

### Construction of a β-Carotene Biosynthesis Pathway and Enhancement of β-Carotene Production in *Y. lipolytica*

**FIGURE 1:**
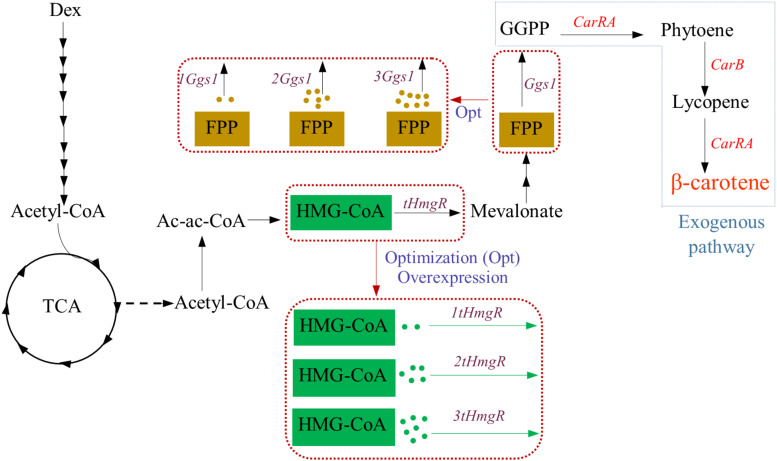
Strategy for β-carotene biosynthesis pathway used in this study. Dex, dextrose; G3P, glyceraldehyde-3-phosphate; AcCoA, Acetyl-CoA; HMG-CoA, hydroxymethylglutaryl-CoA; FPP, farnesyl diphosphate; GGPP, geranylgeranyl diphosphate; tHMG, truncated hydroxymethylglutaryl-CoA reductase; GGS1, GGPP synthase; CarRA, phytoene synthase/lycopene cyclase; CarB, phytoene dehydrogenase.

Strategies used to incorporate the β-carotene biosynthesis pathway in *Y. lipolytica* used in this study are displayed in Figure 1, respectively. *Y. lipolytica* does not produce β-carotene naturally. However, *Y. lipolytica* is an excellent potential host for β-carotene synthesis because it can provide precursor acetyl-CoA and has lipid bodies to store the β-carotene. To achieve the β-carotene biosynthesis in *Y. lipolytica*, the introduction of heterogeneous β-carotene biosynthetic genes is indispensable. Thus, the genes *CarRA* and *CarB* from *B. trispora* were introduced into *Y. lipolytica*, and these two genes were codon-adapted for better expression.

The integration of DNA fragments into the genome of *Y. lipolytica* involves homologous recombination (HR). However, non-homologous end joining (NHEJ) is much more frequent than HR in *Y. lipolytica* because the *Ku70* gene plays a crucial role in double-strand break repair in the NHEJ pathway (Jonathan et al., 2013). Thus, the disruption of the *Ku70* gene significantly hinders the efficiency of NHEJ and improves that of HR. In this study, the *Ku70* gene was deleted to facilitate the integration of large DNA fragments, and all the engineered strains were constructed based on the *Y. lipolytica* po1f*-*Δ*Ku70*.

The codon-adapted *CarRA* and *CarB* from *B. trispora* were incorporated into the genome of *Y. lipolytica* po1f*-*Δ*Ku70* to obtain the genetically stable strain YL-C0. Compared to the strain *Y. lipolytica* po1f*-*Δ*Ku70* with native *CarRA* and *CarB* genes, the β-carotene content in the YL-C0 strain was increased from 0 to 0.6 mg/g DCW. Given the low content of β-carotene in the YL-C0 strain, the genetically stable strain YL-C1 was constructed to increase the β-carotene content by integrating *tHmgR* and *Ggs1* into the genome of YL-C0, and the content of β-carotene in engineered strains YL-C1 was 2.3 mg/g DCW (Supplementary Figure 1). To further increase the content of β-carotene, we decided to increase the expression level of tHMGR and GGS1 because an appropriate expression level can reduce the accumulation of intermediates to increase β-carotene production.

### Effect of Rate-Limiting Enzyme tHMGR on β-Carotene and HMG-CoA Content

**FIGURE 2:**
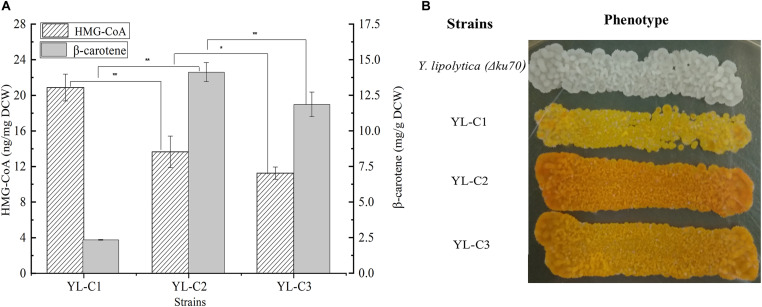
HMG-CoA and β-carotene content in different engineered β-carotene strains. **(A)** HMG-CoA content (ng/mg DCW), β-carotene content (mg/g DCW) in the strains YL-C1, YL-C2, and YL-C3. **(B)** Strains YL-C1, YL-C2, and YL-C3 growing on YPD-agar plate where the color provoked by the β-carotene can be seen. ^∗^*p* < 0.05, and ^∗∗^*p* < 0.01.

truncated HMGR is the key rate-limiting enzyme in the β-carotene synthesis pathway, and it mainly converts HMG-CoA to mevalonate. The inadequate expression of tHMGR causes the HMG-CoA to accumulate. Therefore, the HMG-CoA flow to β-carotene decreases, which reduces the β-carotene production. This study improved β-carotene production by increasing the tHMGR expression to minimize the HMG-CoA accumulation. We overexpressed one copy of tHMGR (1tHMGR), two copies of tHMGR (2tHMGR), and three copies of tHMGR (3tHMGR) in the cell, including the native HMGR on the chromosome, resulting in the engineered strains YL-C1, YL-C2, and YL-C3, respectively. The HMG-CoA content and β-carotene were measured. As shown in Figure 2A, HMG-CoA gradually decreased with the increase of the expression level of tHMGR. Notably, the HMG-CoA content was significantly reduced and reached 13.6 ng/mg DCW when there were 2tHMGR. Moreover, the content of HMG-CoA slightly decreased when there were 3tHMGR. Figure 2A also showed that the content of β-carotene reached the highest 14.1 mg/g DCW when there were 2tHMGR. The color change of the engineered strain was shown in Figure 2B, and the color of the strain was the darkest when there were 2tHMGR. These results indicate that increasing the expression level of tHMGR promoted the conversion of HMG-CoA to β-carotene. Furthermore, the overexpression of 2tHMGR genes is considered appropriate in engineered β-carotene producing strains.

### Effect of Rate-Limiting Enzyme GGS1 on β-Carotene and FPP Content

**FIGURE 3:**
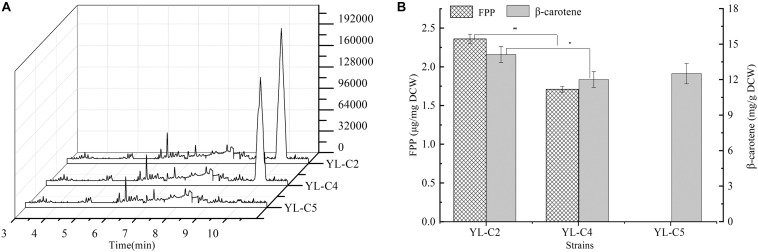
FPP and β-carotene content in different engineered β-carotene strains. **(A)** GC-MS total ions chromatogram of the FPP derivative. **(B)** The FPP content (μg/mg DCW), β-carotene content (mg/g DCW) in the strains YL-C2, YL-C4, and YL-C5. ^∗^*p* < 0.05, and ^∗∗^*p* < 0.01.

GGS1 is another key rate-limiting enzyme in the β-carotene biosynthesis pathway, and it converts the FPP to GGPP. Inadequate GGS1 expression leads to the accumulation of FPP. Therefore, the FPP flow to β-carotene decreases, which reduces the β-carotene production. This study improved β-carotene production by increasing GGS1 expression to minimize FPP accumulation. We overexpressed 1GGS1, 2GGS1, and 3GGS1 in the cell, including the native GGS1 on the chromosome, resulting in the engineered strains YL-C2, YL-C4, and YL-C5, respectively. The FPP and β-carotene content were measured. The GC-MS total ions chromatogram of the FPP derivative in different engineered strains and corresponding retention times (RT 10.085 min) are shown in Figure 3A. Figures 3A,B show that FPP gradually decreased with the increase of GGS1 expression level. Moreover, the concentration of FPP was below the detection level when there were 3GGS1. Meanwhile, the β-carotene content was 12.5 DCW mg/g (Figure 3B). This result suggests that increasing the expression level of rate-limiting enzyme GGS1 contributed to FPP conversion to GGPP synthesis. Furthermore, it is appropriate to overexpress 3GGS1 genes in the engineered β-carotene production strain.

### Transcriptional Level of Related Genes in the β-Carotene Biosynthesis Pathway

**FIGURE 4:**
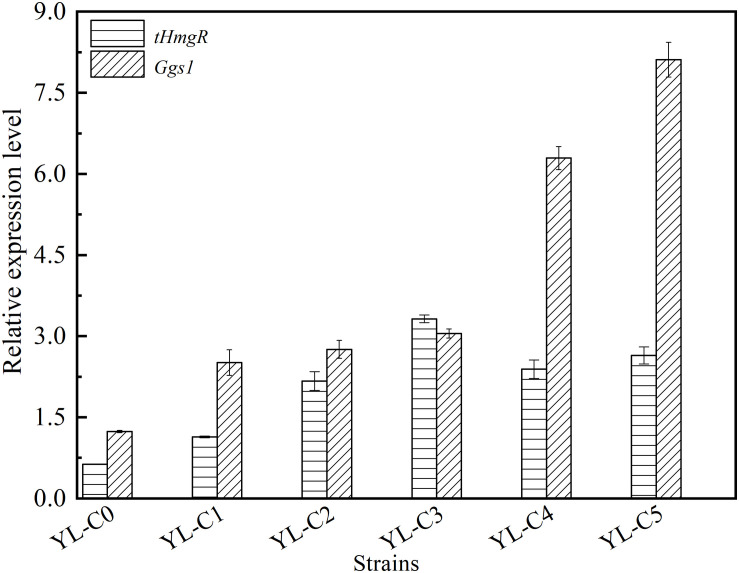
Relative expression levels of *tHmgR*, *Ggs1*, *CarRA*, and *CarB* genes in different engineered β-carotene strains.

The copy number of *tHmgR* was two when the accumulation of intermediate HMG-CoA is the least. The copy number of *tHmgR* was three when the accumulation of intermediate FPP is the least. The difference in copy numbers of *tHmgR* and *Ggs*1, most likely, may have caused the difference in their transcriptional level. The copy numbers of *tHmgR* and *Ggs1* are different when the accumulation of intermediates HMG-CoA and FPP are the least, which may be caused by the difference of their transcriptional level. So we measured and analyzed the transcription level of *tHmgR* and *Ggs1* in control YL-C0, YL-C1 (1*tHmgR*, 1*Ggs1*, 1*CarRA*, 1*CarB*), YL-C2 (2*tHmgR*, 1*Ggs1*, 1*CarRA*, 1*CarB*), YL-C3 (3*tHmgR*, 1*Ggs1*, 1*CarRA*, 1*CarB*), YL-C4 (2*tHmgR*, 2*Ggs1*, 1*CarRA*, 1*CarB*), and YL-C5 (2*tHmgR*, 3*Ggs1*, 1*CarRA*, 1*CarB*) strains. The results were normalized using the *Actin* as the internal standard. As shown in Figure 4, the transcriptional levels of *tHmgR*, *Ggs1* in control YL-C0 were 0.6 and 1.2, respectively, and achieved 3.3 and 8.1 in the strain YL-C5 with the least intermediate. The transcription level of *tHmgR* was lower than that of *Ggs1*. Based on this trend, the optimal copy number of *tHmgR* should be higher than that of the *Ggs1* in the YL-C5 strain. However, the previous experimental results (Figures 2, 3) showed that the copy number of *tHmgR* was lower than that of the *Ggs1* in the engineered strain YL-C5. We speculate that this phenomenon may be caused by the enzyme activity, and the enzyme activity of truncated hydroxymethylglutaryl-CoA reductase is higher than the enzyme activity of GGPP synthase. Therefore, copy numbers of *tHmgR* are lower than those of *Ggs1* when HMG-CoA and FPP accumulation are the least.

### Effect of Rate-Limiting Enzyme CarRA and CarB on HMG-CoA, FPP, and β-Carotene Content

**FIGURE 5:**
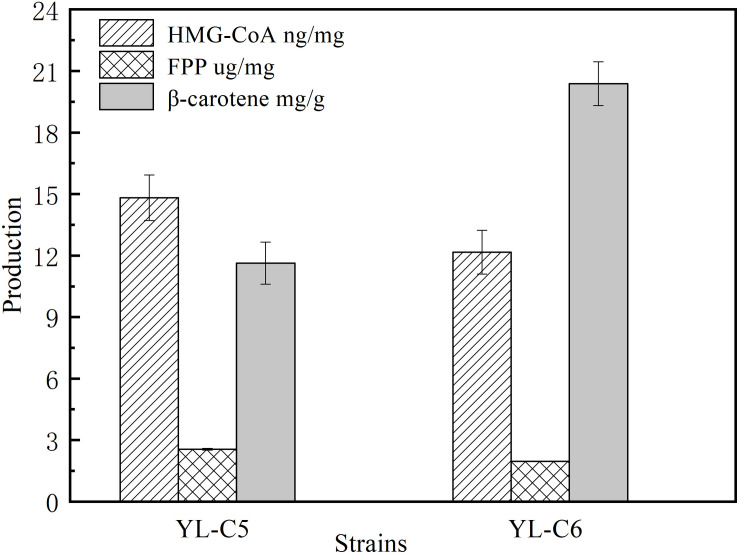
FPP, HMG-CoA, and β-carotene content in different engineered β-carotene strains.

CarRA and CarB also are the important rate-limiting enzymes of the β-carotene biosynthesis pathway. CarRA mainly catalyzes the synthesis of GGPP to phytoene and lycopene to β-carotene, and CarB catalyzes the synthesis of phytoene to lycopene. To further improve the content of β-carotene by minimizing the accumulation of intermediates, we constructed the genetically stable YL-C6 (2*tHmgR*, 3*Ggs1*, 2*CarRA*, 2*CarB*). The content of intermediates and β-carotene were measured in YL-C5 and YL-C6. As shown in Figure 5, compared with YL-C5, the content of HMG-CoA decreased by 15%, and the content of FPP was reduced by 2.3% in engineered strain YL-C6. These results indicate that increased expression levels of CarRA and CarB slightly reduced the accumulation of intermediates HMG-CoA and FPP. Simultaneously, further enhancement of CarRA and CarB’s expression levels may decrease the phytoene and lycopene in engineered strain YL-C6. Notably, the β-carotene content increased by 75.2% to 21.6 mg/g DCW in strain YL-C6 compared with that in YL-C5 after 96 h fermentation (Figure 5). These results indicate that increased expression levels of CarRA and CarB helped the downstream intermediates flow to the β-carotene, and the YL-C6 is the most-productive strain.

### β-Carotene Production in Bioreactor Fermentation

**FIGURE 6:**
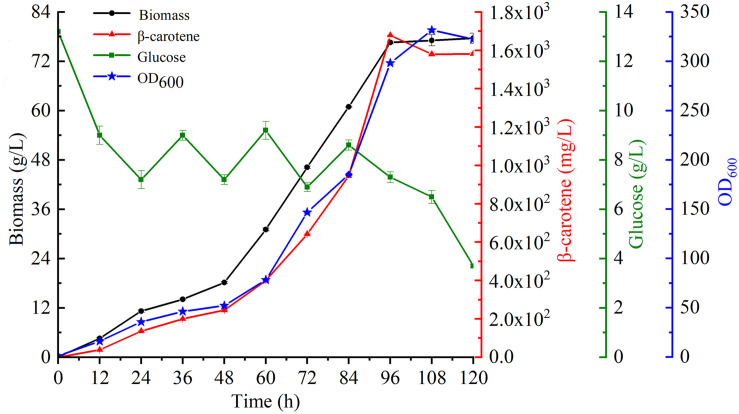
Biomass, β-carotene content, glucose consumption, and OD_600_ of YL-C6 in fermentation.

To further characterize β-carotene production in the most productive strain YL-C6, fermentation was conducted using a 5-L stirred-tank bioreactor. The glucose concentration was maintained at a low level by feeding essential nutrients incrementally to sustain biomass and enhance productivity. Strikingly, the biomass of engineered strain YL-C6 quickly accumulated: the DCW reached 77.6 g/L over 120 h of fermentation, and a maximum of 1.7 g/L of β-carotene was produced after 96 h (Figure 6). These results demonstrate the great potential for the industrial production of β-carotene using metabolic engineered *Y. lipolytica*.

## Discussion

*Yarrowia lipolytica* is currently metabolically engineered to produce several compounds such as fatty acids, terpenoids, and β-carotene (Cao et al., 2016; Larroude et al., 2017; Liu et al., 2019). The biosynthesis of β-carotene in *Y. lipolytica* is a complex process. Selecting and expressing the appropriate heterologous genes alone are not sufficient to accomplish the desired outcome. Constructing codon-adapted heterologous genes and maintaining the metabolic balance of metabolic pathways are two critical strategies for increasing target product yield.

### The Selection of Excellent Genes and the Construction of Codon-Adapted Genes Are Critical for the Target Product Synthesis

The expression of heterologous genes in organisms has become an effective strategy for the synthesis of natural products. The microorganisms can produce more target product when integrating excellent heterologous genes into microorganisms than the mediocre genes. The integration of the genes from *X. dendrorhous* into *S. cerevisiae* increased the lycopene content by 1.2 mg/g compared to the same genes from *Erwinia uredovora* (Bahieldin et al., 2014; Xie et al., 2015). The expression of the genes from *Staphylococcus aureus* in *E. coli* increased the amorphadiene content by 244-fold compared to the gene from *S. cerevisiae* (Vincent et al., 2003; Tsuruta et al., 2009). The above mentioned phenomenon may be caused by a higher catalytic activity of the enzymes encoded by the genes from *X. dendrorhous* and *S. aureus* compared to the same enzymes encoded by the genes from *E. uredovora* and *S. cerevisiae*. In this study, the codon-adapted *CarRA* and *CarB* genes were integrated into the genome of *Y. lipolytica*, which accumulated β-carotene constitutively levels up to 2-fold higher than that of the *Y. lipolytica* expressing the same genes from *M. circinelloides* (Gao et al., 2017a, b). This result indicated that the enzymes CarRA and CarB from *B. trispora* has higher catalytic activity than the same enzymes from *M. circinelloides* (Gao et al., 2014; Yin et al., 2017). Therefore, the selection of enzymes CarRA and CarB from *B. trispora* with high catalytic and construction of codon-adapted *CarRA* and *CarB* are critical for the β-carotene synthesis in *Y. lipolytica*.

### Metabolic Balance Is Crucial for Increasing Target Product Production

The maintenance of metabolic balance at the connecting node is critical because flux balance in metabolic pathways often reduces the accumulation of cytotoxic intermediates and increases target product synthesis. Regulating the metabolic balance by increasing the expression levels of key rate-limiting enzymes to minimize intermediates’ accumulation is crucial for achieving the highest target product content. In the β-carotene biosynthetic pathway of *Y. lipolytica*, tHMGR, GGS1, CarRA, and CarB are the four rate-limiting enzymes in the pivotal connecting node. tHMGR catalyzes the synthesis of HMG-CoA to mevalonate, and GGS1 catalyzes the conversion of FPP to GGPP. CarRA mainly catalyzes the synthesis of GGPP to phytoene and lycopene to β-carotene, and CarB catalyzes the synthesis of phytoene to lycopene. The HMG-CoA, FPP, GGPP, lycopene, and phytoene are the important precursors for β-carotene synthesis. The inadequate expression of tHMGR GGS1, CarRA, and CarB cause HMG-CoA FPP, GGPP, lycopene and phytoene to accumulate, which decreases the supply of β-carotene precursors. In this study, we overexpressed the genes *tHmgR, Ggs1, CarRA*, and *CarB* with *Snf, Lip1, Pox3*, and *Pox4* as the target sites, which caused the deletion of the genes *Snf, Lip1, Pox3*, and *Pox4*. The lipid body formation could be increased remarkably when *Snf, Lip1, Pox3*, and *Pox4* genes were deleted (Dulermo and Nicaud, 2011; Seip et al., 2013). The increase of lipid body can provide enough storage space for β-carotene storage. Increasing the copy number of tHMGR to two achieved the minimal accumulation of HMG-CoA, and simultaneously the β-carotene content increased by 6-fold in the engineered strain YL-C2. The increase of GGS1 copy number to three, the concentration of FPP was below the detection level in the engineered strain YL-C5. Subsequently, elevating the expression levels of *CarRA* and *CarB* almost minimized the accumulation of intermediates (GGPP, phytoene, and lycopene) and increased the β-carotene content by 75.2%. The above phenomena indicate that the overexpression of key rate-limiting enzymes facilitated the intermediate catalysis in the β-carotene biosynthetic pathway, which accelerated the accumulation of β-carotene. The lycopene production was increased by 80% when increasing the copy number of *CrtI* reduced the accumulation of intermediate carotenoids (Xie et al., 2015). The highest isoprene production was obtained by overexpressing MVD1 and IDI1 to promote the conversion of intermediates IPP/DMAPP (Yao et al., 2018). The lycopene production was increased by 1.8-fold when increasing the copy number of *CrtI* reduced phytoene accumulation (Schwartz et al., 2017), These phenomena are also consistent with the results of this study. Therefore, the regulation of the pivotal connecting node’s metabolic balance by minimizing the accumulation of intermediates is crucial for increasing the content of β-carotene.

Furthermore, in oleaginous yeast *Y. lipolytica*, β-carotene biosynthetic pathway competes with the lipid biosynthetic pathway for the same precursor, acetyl- CoA. Increasing the expression of key rate-limiting enzymes (tHMGR GGS1, CarRA, and CarB) in the β-carotene biosynthetic pathway accelerates acetyl-CoA flow to the β-carotene, which decreases precursor supply to the lipid biosynthetic pathway. The inadequate supply of precursors reduces lipid production. Since β-carotene is a liposoluble pigment, the lipid body provides storage space for β-carotene. Therefore, there is a flux balance between lipid biosynthesis and β-carotene biosynthetic pathway.

In summary, the strategies of constructing codon-adapted heterologous genes and maintaining metabolic balance by minimizing the accumulation of intermediates have guided us to achieve a maximal β-carotene content, 1.7 g/L. These successful engineering strategies also provide a foundation for the large-scale production of other products. Regulating metabolic balance by minimizing the accumulation of intermediates also provides a new strategy for engineering bacteria or combinatorial biology to quickly establish metabolic balance.

## Data Availability Statement

The original contributions presented in the study are included in the article/Supplementary Material, further inquiries can be directed to the corresponding author.

## Author Contributions

LL, YQ, GD, and YM were involved in the conception and design of the study. LL and YQ carried out the experiment. JW helped with the data analyses. LL, CH, and YM wrote the manuscript. All authors read and approved the final manuscript.

## Conflict of Interest

The authors declare that the research was conducted in the absence of any commercial or financial relationships that could be construed as a potential conflict of interest.

## Footnotes

1 http://www.kazusa.or.jp/codon

## References

[B1] Ajikumar P. K. Tyo K. Carlsen S. Mucha O. Heng P. T. Stephanopoulos G. (2008). Terpenoids: opportunities for biosynthesis of natural product drugs using engineered microorganisms. *Mol. Pharm.* 5 167–190. 10.1021/mp700151b 18355030

[B2] Bahieldin A. Gadalla N. O. Al-Garni S. M. Almehdar H. Noor S. Hassan S. M. (2014). Efficient production of lycopene in *Saccharomyces cerevisiae* by expression of synthetic crt genes from a plasmid harboring the ADH2 promoter. *Plasmid* 72 18–28. 10.1016/j.plasmid.2014.03.001 24680933

[B3] Cao X. Lv Y. Chen J. Imanaka T. Wei L. Hua Q. (2016). Metabolic engineering of oleaginous yeast *Yarrowia lipolytica* for limonene overproduction. *Biotechnol. Biofuels* 9:214. 10.1186/s13068-016-0626-7 27777617 PMC5057495

[B4] Das A. Yoon S. H. Lee S. H. Kim J. Y. Oh D. K. Kim S. W. (2007). An update on microbial carotenoid production: application of recent metabolic engineering tools. *Appl. Microbiol. Biotechnol.* 77:505. 10.1007/s00253-007-1206-3 17912511

[B5] Donald G. Hampton R. Y. Fritz B. I. (1997). Effects of overproduction of the catalytic domain of 3-hydroxy-3-methylglutaryl coenzyme A reductase on squalene synthesis in *Saccharomyces cerevisiae*. *Appl. Environ. Microbiol.* 63 3341–3344. 9292983 10.1128/aem.63.9.3341-3344.1997 PMC168639

[B6] Dulermo T. Nicaud J. M. (2011). Involvement of the G3P shuttle and β-oxidation pathway in the control of TAG synthesis and lipid accumulation in *Yarrowia lipolytica*. *Metab. Eng.* 13 482–491. 21620992 10.1016/j.ymben.2011.05.002

[B7] Fickers P. Le D. M. Gaillardin C. P. Nicaud J. M. (2003). New disruption cassettes for rapid gene disruption and marker rescue in the yeast *Yarrowia lipolytica*. *J. Microbiol. Methods* 55 727–737. 10.1016/j.mimet.2003.07.003 14607415

[B8] Gao S. Han L. Zhu L. Ge M. Yang S. Jiang Y. (2014). One-step integration of multiple genes into the oleaginous yeast *Yarrowia lipolytica*. *Biotechnol. Lett.* 36 2523–2528. 10.1007/s10529-014-1634-y 25216641

[B9] Gao S. Tong Y. Zhu L. Ge M. Jiang Y. Chen D. (2017a). Production of beta-carotene by expressing a heterologous multifunctional carotene synthase in *Yarrowia lipolytica*. *Biotechnol. Lett.* 39 921–927. 10.1007/s10529-017-2318-1 28289912

[B10] Gao S. Tong Y. Zhu L. Ge M. Zhang Y. Chen D. (2017b). Iterative integration of multiple-copy pathway genes in *Yarrowia lipolytica* for heterologous β-carotene production. *Metab. Eng.* 41 192–201. 10.1016/j.ymben.2017.04.004 28414174

[B11] Huang B. Zeng H. Dong L. Li Y. Sun L. Zhu Z. (2011). Metabolite target analysis of isoprenoid pathway in *Saccharomyces cerevisiae* in response to genetic modification by GC-SIM-MS coupled with chemometrics. *Metabolomics* 7 134–146. 10.1007/s11306-010-0240-9

[B12] Jon A. F. Victor W. R. (2004). The 3-hydroxy-3-methylglutaryl coenzyme-A (HMG-CoA) reductases. *Genome Biol.* 5:248. 10.1186/gb-2004-5-11-248 15535874 PMC545772

[B13] Jonathan V. Athanasios B. Jean-Marc N. (2013). Efficient homologous recombination with short length flanking fragments in *Ku70* deficient *Yarrowia lipolytica* strains. *Biotechnol. Lett.* 35 571–576. 10.1007/s10529-012-1107-0 23224822

[B14] Larroude M. Celinska E. Back A. Thomas S. Nicaud J. M. Ledesma A. R. (2017). A synthetic biology approach to transform *Yarrowia lipolytica* into a competitive biotechnological producer of β-carotene: production of β-carotene in *Y. lipolytica*. *Biotechnol. Bioeng.* 115 464–472. 10.1002/bit.26473 28986998

[B15] Lee P. Schmidt-Dannert C. (2002). Metabolic engineering towards biotechnological production of carotenoids in microorganisms. *Appl. Microbiol. Biotechnol.* 60 1–11. 10.1007/s00253-002-1101-x 12382037

[B16] Liu L. You Y. Deng H. Guo Y. Meng Y. (2019). Promoting hydrolysis of apple pomace by pectinase and cellulase to produce microbial oils using engineered *Yarrowia lipolytica*. *Biomass Bioenergy* 126 62–69. 10.1016/j.biombioe.2019.04.025

[B17] Matthäus F. Ketelhot M. Gatter M. Barth G. (2014). Production of lycopene in the non-carotenoid-producing yeast *Yarrowia lipolytica*. *Appl. Environ. Microbiol.* 80 1660–1669. 10.1128/AEM.03167-13 24375130 PMC3957620

[B18] Mehta B. J. Obraztsova I. N. Cerda O. E. (2003). Mutants and intersexual heterokaryons of *Blakeslea trispora* for production of β-Carotene and lycopene. *Appl. Environ. Microbiol.* 69 4043–4048. 10.1128/AEM.69.7.4043-4048.2003 12839780 PMC165160

[B19] Nanou K. Roukas T. (2016). Waste cooking oil: a new substrate for carotene production by *Blakeslea trispora* in submerged fermentation. *Bioresour. Technol.* 203 198–203. 10.1016/j.biortech.2015.12.053 26724551

[B20] Olson M. L. Johnson J. Carswell W. F. Reyes L. H. Senger R. S. Kao K. C. (2016). Characterization of an evolved carotenoids hyper-producer of *Saccharomyces cerevisiae* through bioreactor parameter optimization and Raman spectroscopy. *J. Ind. Microbiol. Biotechnol.* 43 1355–1363. 10.1007/s10295-016-1808-9 27423881

[B21] Schwartz C. Frogue K. Misa J. Wheeldon I. (2017). Host and pathway engineering for enhanced lycopene biosynthesis in *Yarrowia lipolytica*. *Front. Microbiol.* 8:2233. 10.3389/fmicb.2017.02233 29276501 PMC5727423

[B22] Seip J. Jackson R. He H. X. Zhu Q. Hong S. P. (2013). Snf1 is a regulator of lipid accumulation in *Yarrowia lipolytica*. *Appl. Environ. Microbiol.* 79 7360–7370. 24056466 10.1128/AEM.02079-13 PMC3837744

[B23] Su A. Chi S. Li Y. Tan S. Qiang S. Chen Z. (2018). Metabolic redesign of *Rhodobacter sphaeroides* for lycopene production. *J. Agric. Food Chem.* 66 5879–5885. 10.1021/acs.jafc.8b00855 29806774

[B24] Tsuruta H. Paddon C. J. Eng D. Lenihan J. R. Horning T. Anthony L. C. (2009). High-level production of amorpha-4,11-diene, a precursor of the antimalarial agent artemisinin, in *Escherichia coli*. *PLoS One* 4:e4489. 10.1371/journal.pone.0004489 19221601 PMC2637983

[B25] Verwaal R. Wang J. Meijnen J. P. Visser H. Sandmann G. van den Berg J. A. (2007). High-level production of beta-carotene in *Saccharomyces cerevisiae* by successive transformation with carotenogenic genes from *Xanthophyllomyces dendrorhous*. *Appl. Environ. Microbiol.* 73 4342–4350. 10.1128/AEM.02759-06 17496128 PMC1932764

[B26] Vincent J. J. M. Douglas J. P. Sydnor T. W. Jack D. N. Keasling J. D. (2003). Engineering a mevalonate pathway in *Escherichia coli* for production of terpenoids. *Nat. Biotechnol.* 21 796–802. 12778056 10.1038/nbt833

[B27] Wang G. Xiong X. Ghogare R. Wang P. Meng Y. Chen S. (2016). Exploring fatty alcohol-producing capability of *Yarrowia lipolytica*. *Biotechnol. Biofuels* 9:107. 10.1186/s13068-016-0512-3 PMC4875687 27213014

[B28] Wu T. Ye L. Zhao D. Li S. Li Q. Zhang B. (2017). Membrane engineering-A novel strategy to enhance the production and accumulation of β-carotene in *Escherichia coli*. *Metab. Eng.* 43 85–91. 10.1016/j.ymben.2017.07.001 28688931

[B29] Xie W. Lv X. Ye L. Zhou P. Yu H. (2015). Construction of lycopene-overproducing *Saccharomyces cerevisiae* by combining directed evolution and metabolic engineering. *Metab. Eng.* 30 69–78. 10.1016/j.ymben.2015.04.009 25959020

[B30] Yao Z. Zhou P. Su B. Su S. Ye L. Yu H. (2018). Enhanced isoprene production by reconstruction of metabolic balance between strengthened precursor supply and improved isoprene synthase in *Saccharomyces cerevisiae*. *ACS Synth. Biol.* 7 2308–2316. 10.1021/acssynbio.8b00289 30145882

[B31] Yen H. Liao Y. Yi X. (2015). The growth of oleaginous *Rhodotorula glutinis* in an airlift bioreactor on crude glycerol through a non-sterile fermentation process. *Bioprocess Biosyst. Eng.* 38 1541–1546. 10.1007/s00449-015-1396-5 25835228

[B32] Yin S. Wu Y. Yang S. Chen S. (2017). Improvement of B-Carotene production in *Yarrowia lipolytica* through assembling multiple copies of B-Carotene biosynthesis genes from *Blakeslea trispora*. *J. Microbiol. Biotechnol.* 6 52–57.

[B33] Yoon S. H. Park H. M. Kim J. E. Lee S. H. Choi M. Kim J. Y. (2007). Increased β-Carotene production in recombinant *Escherichia coli* harboring an engineered isoprenoid precursor pathway with mevalonate addition. *Biotechnol. Prog.* 23 599–605. 10.1021/bp070012p 17500531

[B34] Zhu F. Lu L. Fu S. Zhong X. Hu M. Den Z. (2015). Targeted engineering and scale up of lycopene overproduction in *Escherichia coli*. *Process Biochem.* 50 341–346. 10.1016/j.procbio.2014.12.008

