# Supplementary material for: Elevated β-Carotene Production Using Codon-Adapted CarRA&B and Metabolic Balance in Engineered Yarrowia lipolytica
Source: Front Microbiol. 2021 Mar 4;12:627150. doi: 10.3389/fmicb.2021.627150 (PMC7970187; doi:10.3389/fmicb.2021.627150)

**Supplementary tables**

**Table 1.** Strains and plasmids used in this study

| Strains | description | sources |
| --- | --- | --- |
| *E. coli* DH5α | *fhuA2 Δ*(*argF-lacZ*)U169 *phoA glnV44 Φ80 Δ(lacZ)*M15 *gyrA96 recA1 relA1 endA1 thi-1 hsdR17* | Novagen |
| *Y. lipolytica* po1f | MatA, leucine-, uracil^-^, xpr2-322, axp1-2^-^ | Washington State University Dr. Xiong presented |
| *Y. lipolytica* Po1f*-ΔKu70* | MatA, leucine-, uracil^-^, xpr2-322, axp1-2, *ΔKu70* | This study |
| YL-C0 | MatA, leucine-, uracil^-^, xpr2-322, axp1-2, *ΔKu70 CarRA^+^,CarB^+^* | This study |
| YL-C1 | MatA, leucine-, uracil^-^, xpr2-322, axp1-2, *ΔKu70 tHmgR^+^,Ggs1^+^,CarRA^+^,CarB^+^* | This study |
| YL-C2 | MatA, leucine-, uracil^-^, xpr2-322, axp1-2, *ΔKu70* 2*tHmgR^+^,Ggs1^+^,CarRA^+^,CarB^+^* | This study |
| YL-C3 | MatA, leucine-, uracil^-^, xpr2-322, axp1-2, *ΔKu70* 3*tHmgR^+^,Ggs1^+^,CarRA^+^,CarB^+^* | This study |
| YL-C4 | MatA, leucine-, uracil^-^, xpr2-322, axp1-2, *ΔKu70* 2*tHmgR^+^,*2*Ggs1^+^,CarRA^+^,CarB^+^* | This study |
| YL-C5 | MatA, leucine-, uracil^-^, xpr2-322, axp1-2, *ΔKu70* 2*tHmgR^+^,*3*Ggs1^+^,CarRA^+^,CarB^+^* | This study |
| YL-C6 | MatA, leucine-, uracil^-^, xpr2-322, axp1-2, *ΔKu70* 2*tHmgR^+^,*3*Ggs1^+^,*2*CarRA^+^,*2*CarB^+^* | This study |
| plasmids | used for | sources |
| pLoxp-ura3-Loxp | Knock out vector, loxp, ura3, ura3-testR1, ura3-F, loxp, AmpR | Washington State University Dr. Xiong presented |
| pLoxp-ura3-Loxp-Δ*Ku70* | Knocking out *Ku70*, AmpR | This study |
| pLoxp-ura3-Loxp-Δ*Snf* | Knocking out *Snf*, AmpR | This study |
| pLoxp-ura3-Loxp-Δ*Snf*::optAB | Expressing *CarRA* and *CarB* | This study |
| pLoxp-ura3-Loxp-Δ*Lip1* | Knocking out *Lip1*, AmpR | This study |
| pLoxp-ura3-Loxp-Δ*Lip1*::*tHmgR-Ggs1* | Expressing *tHmgR* and *Ggs1* | This study |
| pLoxp-ura3-Loxp-Δ*Pox3* | Knocking out *Pox3*, AmpR | This study |
| pLoxp-ura3-Loxp-Δ*Pox3*::*tHmgR* | Expressing *tHmgR* | This study |
| pLoxp-ura3-Loxp-Δ*Pox3*::*2tHmgR* | Expressing 2*tHmgR* | This study |
| pLoxp-ura3-Loxp-Δ*Pox3*::*tHmgR-Ggs1* | Expressing *tHmgR* and *Ggs1* | This study |
| pLoxp-ura3-Loxp-Δ*Pox3*::*tHmgR-*2*Ggs1* | Expressing *tHmgR* and 2*Ggs1* | This study |
| *pLoxp-ura3-Loxp-ΔPox4* | Knocking out *Pox4*, AmpR | This study |
| *pLoxp-ura3-Loxp-ΔPox4::optAB* | Expressing *CarRA* and *CarB* | This study |

**Table S2.** List of primers used in this study

| Name | Sequence |
| --- | --- |
| *Ku70-*up-F | ACCGTGGGCCCCATAGGCCCATAAAGTACG |
| *Ku70-*up-R | CAGCTTCTAGATTTCAAAAAGCGGCGGT |
| *Ku70*-down-F | ACTGAACTAGTCTAGGGAGGCACATCTAA |
| *Ku70*-down-R | CTGACCATATGTATCATGGCTGAAGTTGAG |
| *Snf-*up-F | ACTGCGGGCCCGACGCAAAAGGAAGAAACAGA |
| *Snf*-up-R | GTACTTCTAGAGGAGTGGTATGTAGTCGTG |
| *Snf*-down-F | CTGACACTAGTAAACACTCCTTGGTGAACT |
| *Snf*-down-R | CATGACATATGGGAATTCGTGCAGAAGAAC |
| *Snf-*test 1 | GTGGTGGAAGAGACAGAGGT |
| *Snf-*test 2 | CAGATTGCAGCTTTGCCTTC |
| *Ku70*-test 1 | CTTCTTACGACATATACATCAA |
| *Ku70*-test 2 | GTCAGCCTCGCATTGA |
| *Lip1*- up-F | AGCGAGGGCCCTTCACCCGATCAGGCAAGTACAACTC |
| *Lip1-* up-R | GCTGATCTAGAGATTTCTTGACCGGAATTGATAGTTAGTAGACAA |
| *Lip1*- down-F | CGATGACTAGTGGTTCATGAGAAGATAAATATATAAA |
| *Lip1-* down-R | CAGCTCATATGGGTCAAGCATTTCGACCGTCGAATCGACGAGGTG |
| *Lip1*-test 1 | AATCATCGAGAATGGTCTCT |
| *Lip1*-test 2 | CAGCCGCTGAGTACGTTCAT |
| *Pox3*- up-F | TATAGGGGCCCGGCAAATTTTACTGGGGCCTT |
| *Pox3-* up-R | GCTGATCTAGATGTGTGTATCGTAGAGGTAG |
| *Pox3*- down-F | GTACGACTAGTATGGAGCGTGTGTTCTGAGTC |
| *Pox3-* down-R | ACGCACATATGTGTCGAGATATCGACATTGTTC |
| *Pox3*-test 1 | TGTGGGGTATCAAAGCCGAG |
| Pox3-test 2 | TGATATTTGTACTGTTGATA |
| *Pox4*- up-F | TATAGGGGCCCTATGCACCATTAAATGATTA |
| *Pox4-* up-R | GCAGGTCTAGATGTTGTGGGTCGTTTCAATGAA |
| *Pox4*- down-F | GTACGACTAGTTGATTTTGTAGGTTTTAGTAAGTAT |
| *Pox4-* down-R | ACGCACATATGAACGTCCGCGTGTATGTCCCA |
| *Pox4*-test 1 | AACAATATTATTTTGACAGC |
| *Pox4*-test 2 | CAGTTGTCAGCAGCGTGTT |
| Primer-F | GCTAGTCTAGATAGTTTCTTTGTCTGGCCATC |
| Primer-R | GTACGACTAGTAGATCTGAGCGTGAATTATA |
| test F | TCCTGGAGGCAGAAGAACTT |
| test R | ATACGTGAGTCAGAAGGGCT |
| *tHmgR*-F (qPCR) | CTCAGGACGGTATGACACGA |
| *tHmgR*-R (qPCR) | GAGTTGAAGGCCTTTCGCAT |
| *Ggs1-*F (qPCR) | ATCAAGGTGGACAAGAGCGA |
| *Ggs1-*R (qPCR) | CAACAAGCAGCGACGAGTTA |
| *CarRA*-F (qPCR) | GCTCTGCTGGCTATCACCTA |
| *CarRA*-R (qPCR) | GTCGTCGCAGAATGTACTCG |
| *CarB*-F (qPCR) | GACAAGGACAAGCGAGTGAC |
| *CarB*-R (qPCR) | CAGGGTCTTCTTGGTCCAGT |

The procedures to confirm that the expressed gene was integrated into the target site are as follows: The nucleotide sequence was amplified by the test primers of the target gene. The length of the amplified nucleic acid sequence was compared with the known nucleic acid sequence. Finally, the amplified nucleic acid sequence was sequenced to confirm whether the expressed gene has been integrated into the target site.

Figure 1 The biomass, β-carotene content, and production in YL-C0 and YL-C1 strains.


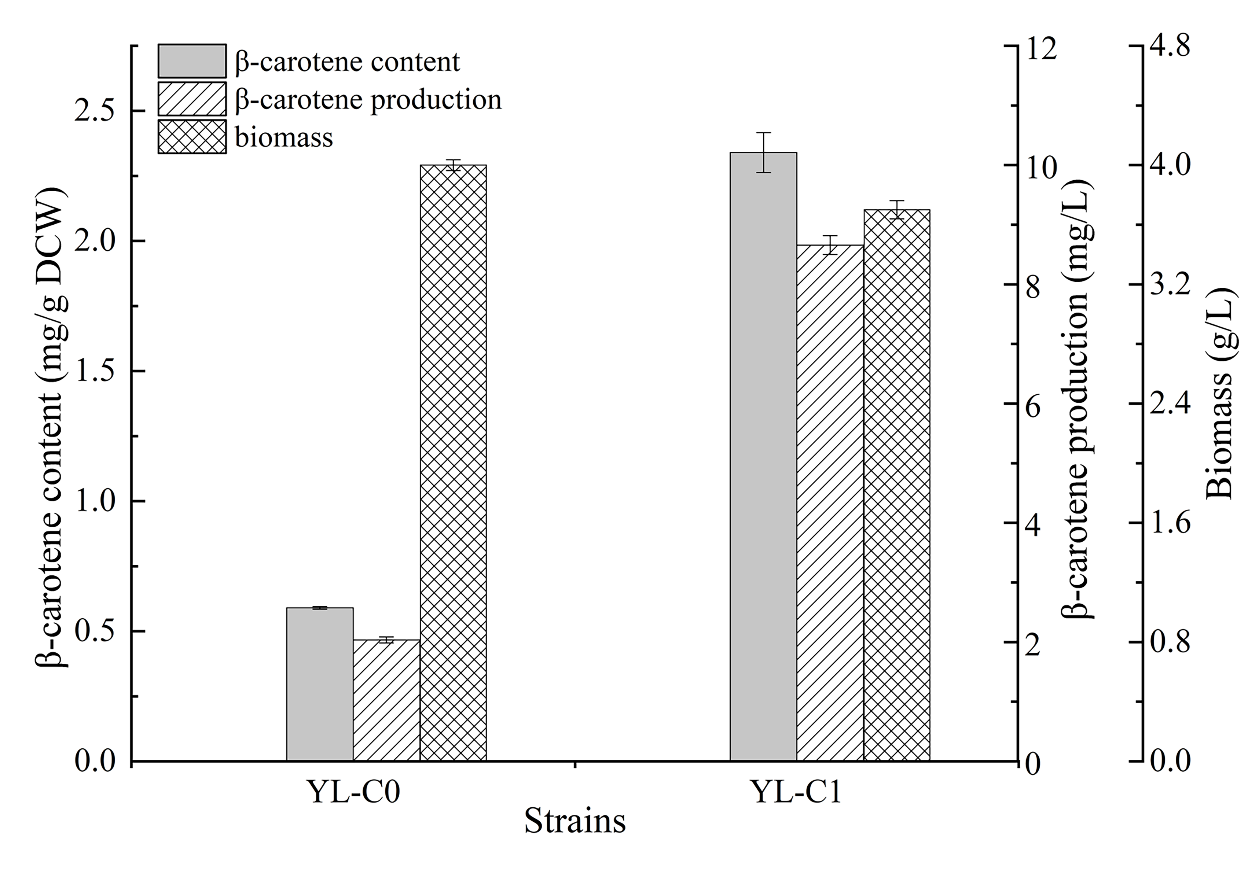


Figure 2a The map of plasmid *Ploxp-ura3-loxp-ΔLip1::tHmgR-Ggs1*


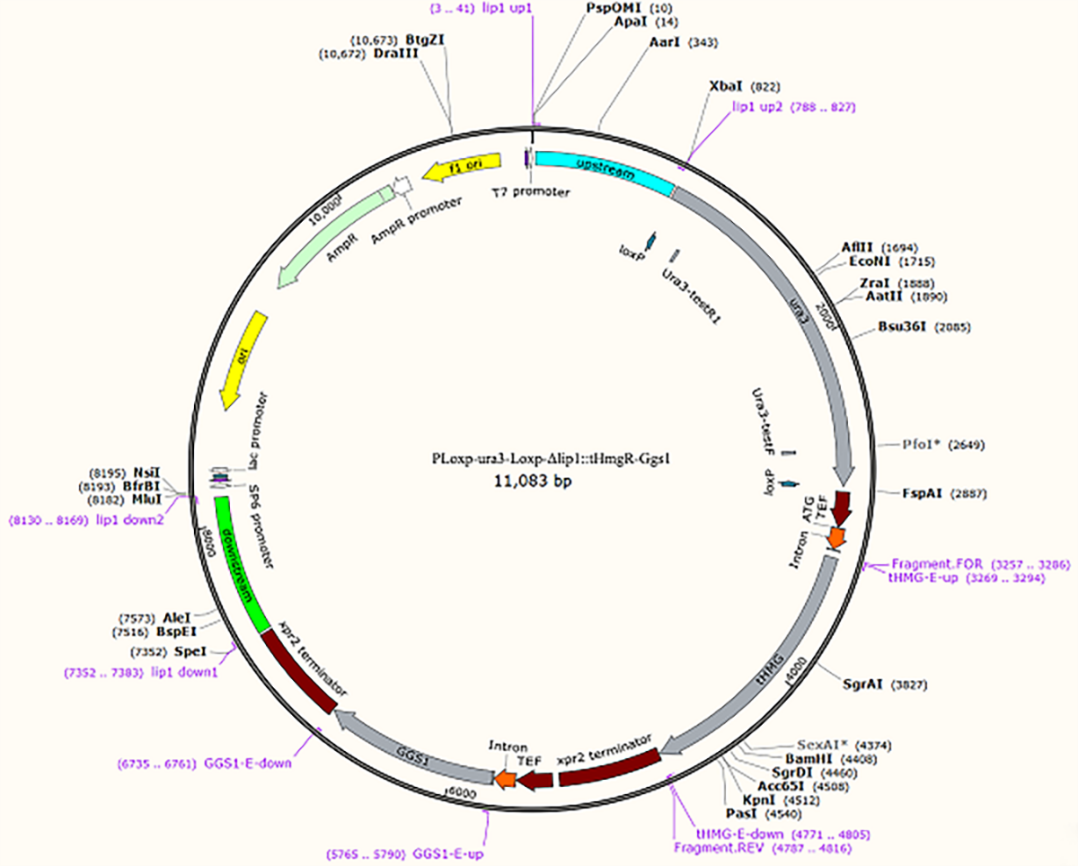


Figure 2b The map of plasmid *Ploxp-ura3-loxp-ΔSnf::optAB*


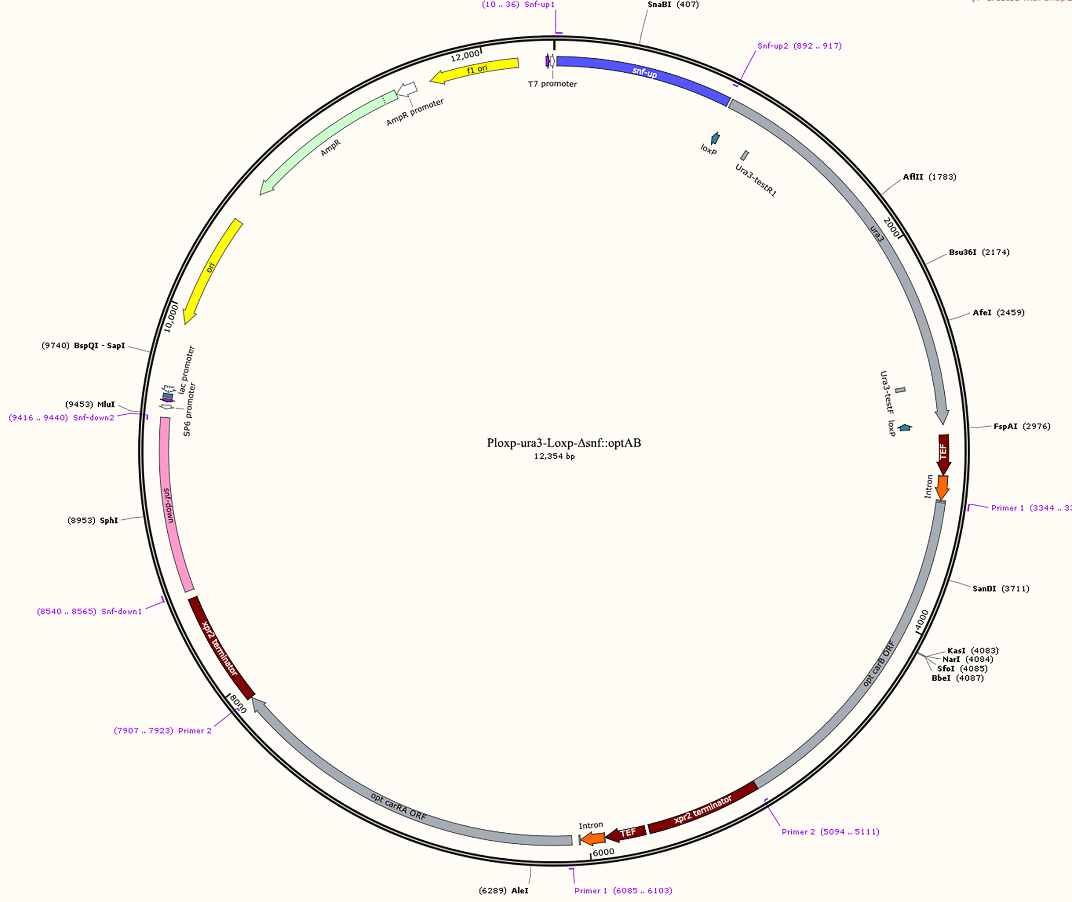


Figure 2c The map of plasmid *Ploxp-ura3-loxp-ΔPox3::2tHmgR*


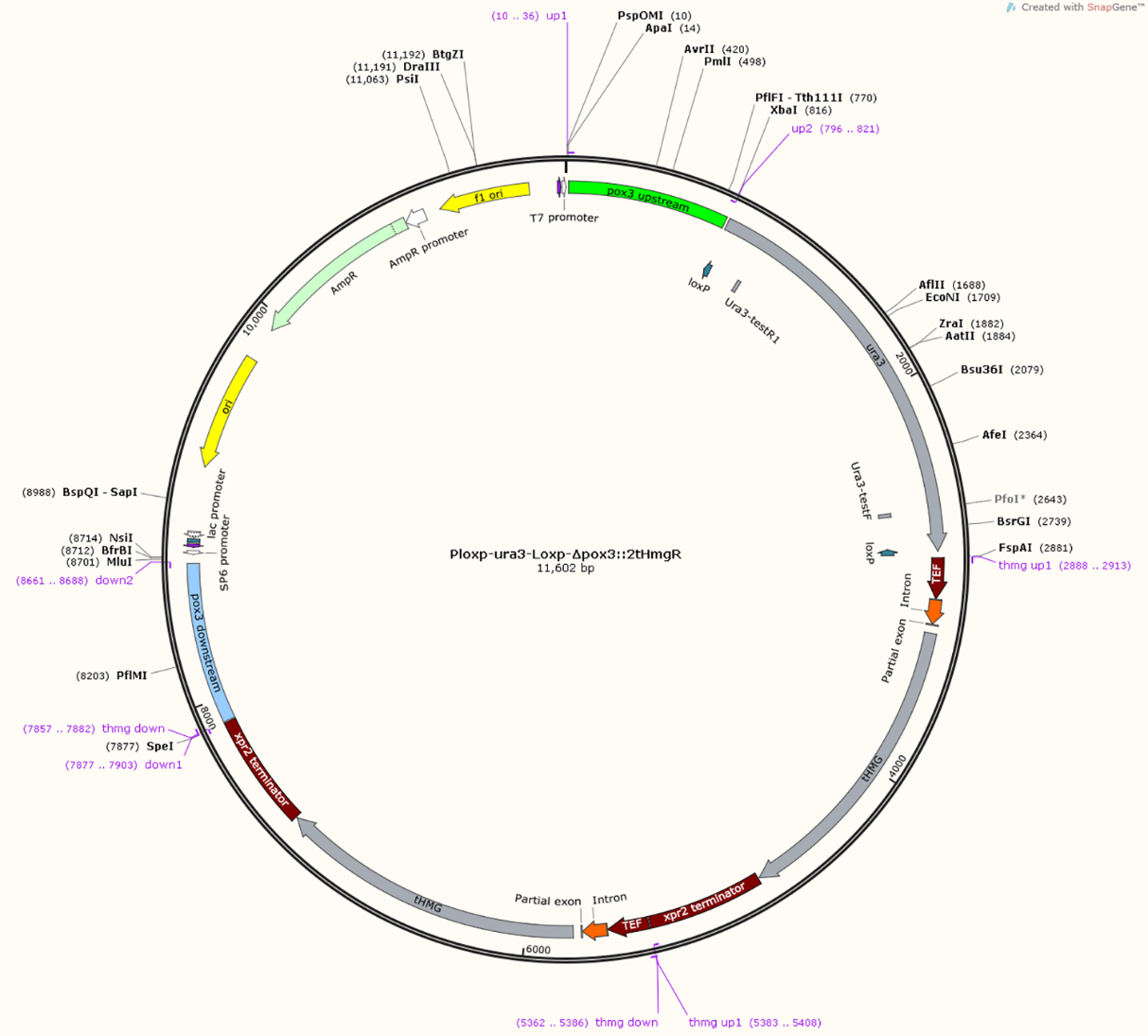


Figure 2d The map of plasmid *Ploxp-ura3-loxp-ΔPox3::tHmgR-2Ggs1*


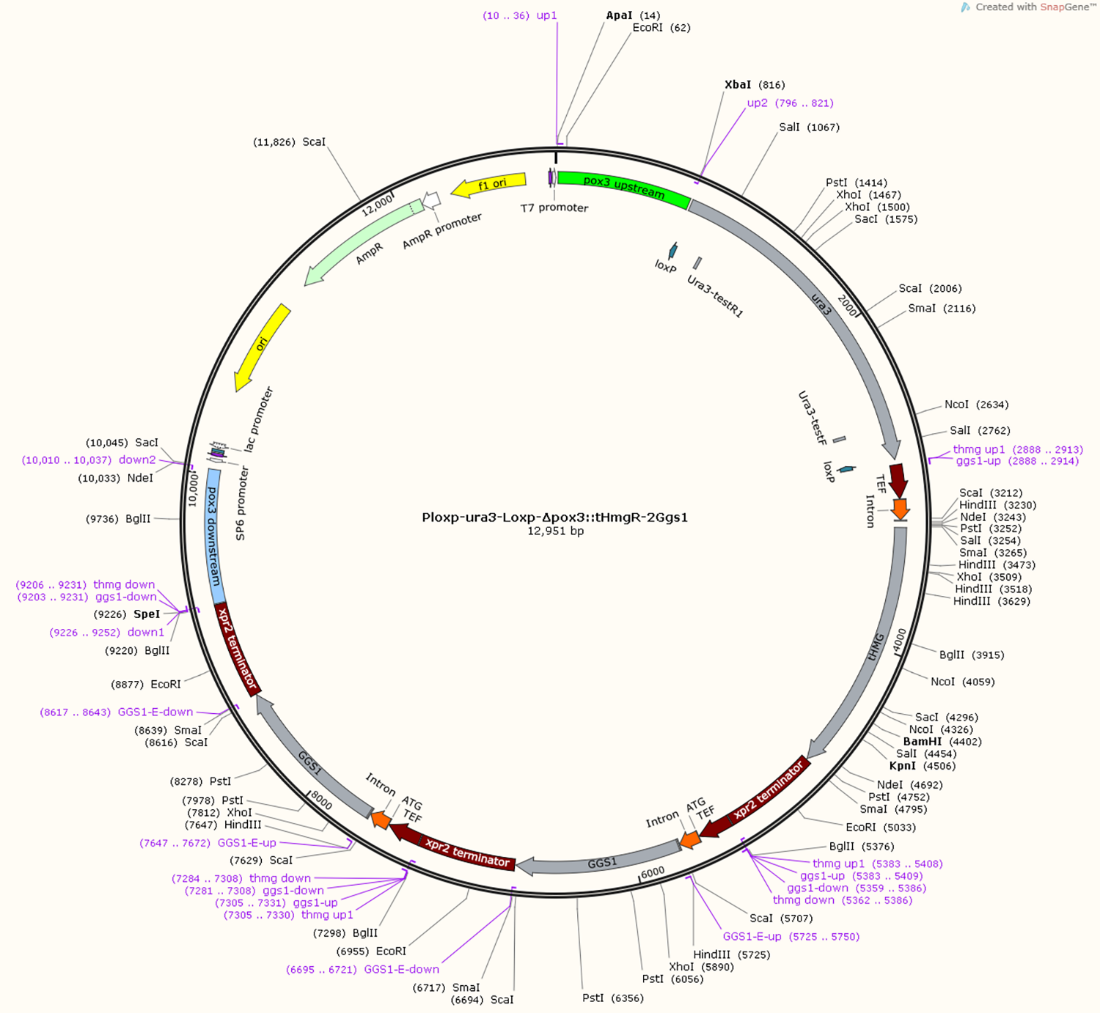

Supplement: Supplementary file 1 [file Data_Sheet_1.docx]
